# Tree Classification with Fused Mobile Laser Scanning and Hyperspectral Data

**DOI:** 10.3390/s110505158

**Published:** 2011-05-11

**Authors:** Eetu Puttonen, Anttoni Jaakkola, Paula Litkey, Juha Hyyppä

**Affiliations:** Department of Photogrammetry and Remote Sensing, Finnish Geodetic Institute, P.O. Box 15 02431 Masala, Finland; E-Mails: anttoni.jaakkola@fgi.fi (A.J.); paula.litkey@fgi.fi (P.L.); juha.hyyppa@fgi.fi (J.H.)

**Keywords:** mobile laser scanning, classification, data fusion, hyperspectrum, forestry

## Abstract

Mobile Laser Scanning data were collected simultaneously with hyperspectral data using the Finnish Geodetic Institute Sensei system. The data were tested for tree species classification. The test area was an urban garden in the City of Espoo, Finland. Point clouds representing 168 individual tree specimens of 23 tree species were determined manually. The classification of the trees was done using first only the spatial data from point clouds, then with only the spectral data obtained with a spectrometer, and finally with the combined spatial and hyperspectral data from both sensors. Two classification tests were performed: the separation of coniferous and deciduous trees, and the identification of individual tree species. All determined tree specimens were used in distinguishing coniferous and deciduous trees. A subset of 133 trees and 10 tree species was used in the tree species classification. The best classification results for the fused data were 95.8% for the separation of the coniferous and deciduous classes. The best overall tree species classification succeeded with 83.5% accuracy for the best tested fused data feature combination. The respective results for paired structural features derived from the laser point cloud were 90.5% for the separation of the coniferous and deciduous classes and 65.4% for the species classification. Classification accuracies with paired hyperspectral reflectance value data were 90.5% for the separation of coniferous and deciduous classes and 62.4% for different species. The results are among the first of their kind and they show that mobile collected fused data outperformed single-sensor data in both classification tests and by a significant margin.

## 1. Introduction

We propose the use of mobile hyperspectral data together with mobile laser-scanning (MLS) data in tree species classification. The Sensei system constructed at the Finnish Geodetic Institute (FGI) integrates laser and hyperspectral data, but it has not yet been demonstrated in practical applications [1]. The rapid development of laser scanning has shifted since the middle of the first decade of the new millennium from airborne and terrestrial systems towards the development of mobile laser scanning, although the concept was first already proposed in 1996 [2]. A MLS system integrates navigation and data acquisition sensors on a rigid moving platform for collecting point clouds from the surroundings of the mapping system. A MLS system is, thus, similar to airborne laser scanning (ALS) systems, but typically applied MLS platforms include a van or a car. An increasing number of MLS systems based on commercial vans or cars are being used in urban and suburban environments, and described by, for example, El-Sheimy [3], and Graham [4]. Several research systems have been introduced recently [e.g., Geomobil (ICC), GeoMaster (University of Tokyo), Lara-3D (Ecoles des Mines de Paris), Roamer (FGI), Sensei (FGI)] [1,5–8]. A large number of studies looking into different types of MLS systems, their accuracies, and suitability for different applications, including environmental modeling, have been published in recent years [4,8–24]. Compared to airborne laser systems, which typically collect point clouds with a resolution of 0.5–40 pts/m^2^ from an altitude of 100–3000 m, MLS provides point clouds with a resolution of hundreds or even thousands of points/m^2^ from a distance of some dozens of meters. ALS and MLS data sets complement each other in several ways as they have different viewing geometries. For example, ALS data provides mainly horizontal structures in urban environments, such as the roofs of buildings, while MLS is at its best in detecting vertical objects, e.g., walls of buildings, trees, and lampposts [15,25,26]. MLS is also used in road line and pavement mapping studies to provide very high resolution spatial surface data [14,17].

In ALS systems, the simultaneous use of laser scanning alone or in combination with multi- or hyperspectral imagery has been studied actively, especially for the purpose of forest assessment and ecological studies [27–43]. ALS is also used for commercial and operative forest inventories especially in Scandinavia and Finland (a.k.a. the Nordic region) where there are only a few dominant and commercially significant tree species [44,45].

In recent years, several ALS-based methods have been developed for single tree species classification in the Nordic region [46–49]. These include the use of laser point cloud shape distributions, full waveform measurements, ALS intensity, computational geometry approach, data fusion with aerial imagery, and their combinations. The presented methods have proved to be successful in dominant tree species classification. However, in temperate regions the species number exceeds ten and the canopy structure is denser and more complex than in Nordic region. Thus, tree species classification, especially on the individual tree level, becomes a significant task. Dalponte *et al.* [30] tested combined hyperspectral imagery and multiple-return ALS data to classify 23 forest classes and obtained over 85% class-wise classification accuracies for dominant classes.

MLS is used in conjunction with urban and suburban photorealistic city models [7,23]. There is also an increasing need to automatically obtain detailed tree data on trees and forests in city areas [50,51]. This is being currently studied using laser point clouds and imaging data obtained from cameras. However, additional image data containing RGB data does not necessarily yield significantly improved data in tree species classification if a relatively dense point cloud is already obtained from each tree and its vertical structure. Thus, in this paper, we deal with simultaneously collected laser point clouds and hyperspectral data from mobile mapping for the purpose of tree species classification and report of the first results. Classification of tree species was performed in three different phases. First, only the spatial data from point clouds were used in classification. Second, only the hyperspectral data from the spectrometer were used in classification. Third, combined data set consisting of both spatial data and hyperspectral data were used in classification.

We found that tree species classification is a challenging task due to the inherent variations in shape and colouring between and within the individual trees of different species. However, the study results also show that the collected MLS data classified 133 tree specimen from 10 different species with over 80% overall classification accuracy in the best tested study case. The result implies that combined MLS data could provide a practical basis for other environmental and urban monitoring and management studies. Furthermore, MLS data has also potential to be applied in a wide range of different applications; for example, in forestry, agriculture, urban planning, and building detection.

## 2. Measurement System, and the Test Area Description

Section 2.1 describes the sensors mounted on the Sensei measurement system. Section 2.2 gives an overview of the test area characteristics, the measurement, and the tree specimen within the test area.

### 2.1. Sensors Mounted on the Sensei Measurement System

**Figure 1.**
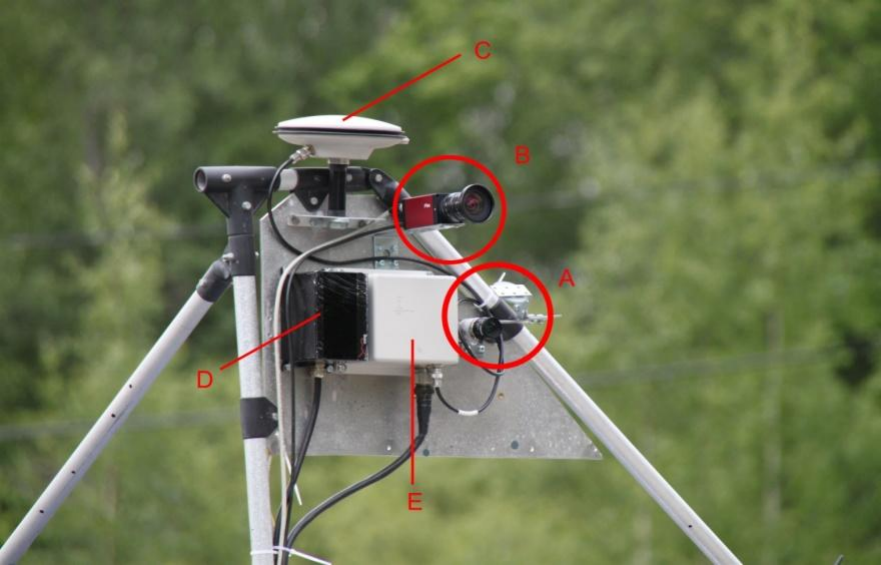
The Sensei measurement system in its mobile mapping configuration mounted on a car. The sensors are as follow: (**A)**. A Specim V10H line spectrometer and a mirror for viewing the Spectralon™ reference panel (not shown in figure); (**B)**. An AVT Pike F-421C CCD camera (not used in this study); (**C)**. A Novatel 702 GG GPS receiver; (**D)**. An Ibeo Lux laser scanner; (**E**). A NovAtel SPAN-CPT Inertial Measurement Unit.

The Finnish Geodetic Institute’s Sensei is a measurement system able to carry a number of different measurement instruments [1]. These include a GPS/IMU positioning system, two laser scanners, a CCD camera, a spectrometer and a thermal camera. The Sensei measurement system has a modular structure, which means that only the instruments required for the specific measurement campaign need to be mounted and new sensors can be easily added to the system. In the present study, data collected with the Ibeo Lux laser scanner (Ibeo Automotive Systems GmbH, Germany), we used the Specim V10H spectral line camera (Spectral Imaging Ltd., Finland), and NovAtel SPAN-CPT inertial navigation system (NovAtel Inc., Canada). Figure 1 shows the measurement system in its mobile mapping configuration.

**Figure 2.**
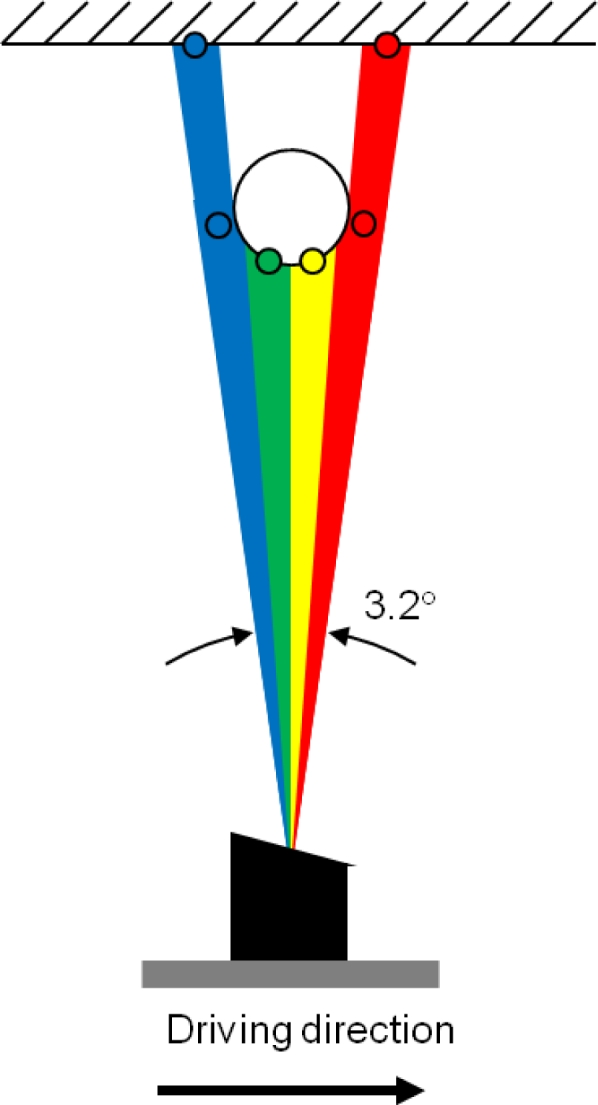
An indicative top-down view schematic of the measurement geometry and principle of the Ibeo Lux laser scanner (not to scale). The different colours show the different layers of the laser beam and point measured by these layers. The colours in figure are not related to hyperspectral data.

The Ibeo Lux scanner measures points from four different layers simultaneously and it has a theoretical scan rate of up to 38,000 points/second if only one return per laser pulse and per layer is assumed. The scanner is able to record up to three returns per laser pulse and per layer. This allows it to get hits from the ground or building walls even when covered by nearby trees or vegetation. Its distance measurement range is from 0.3 to 200 m (50 m for targets with 10% remission), its ranging accuracy is 10 cm, its angular resolution is 0.25°. The divergence of the laser beam is 0.8° horizontally and 0.08° vertically with respect to the scanner body in the current instrument configuration. This indicates that objects may appear extended in the horizontal direction. This elongation caused by the wide footprint of the laser scanner could be mitigated by applying some kind of thinning method to the data. However, as the elongation effect is moderate at short distances, it did not have a significant effect on the classification results. On the other hand, the wide laser beam also allows the laser scanner to cover the target area extensively and acquire points from thin targets as there are no gaps between the layers of the laser scanner. Figure 2 shows an indicative schematic of the measurement geometry.

The Specim V10H spectrometer is a spectral line camera with an instantaneous field of view of about 0.067° and a spectral resolution of 8.5 nm. The spectral range of the spectrometer is 397–1,086 nm and the opening angle of the optics is 44.4° in the vertical direction when mounted on the Sensei system. The spectrometer measures the incoming light by passing it through a diffraction grating to a monochromatic CCD sensor, which produces a line of spectral data with a spatial resolution of 659 pixels and 493 spectral channels. The spectral channels were averaged during the data acquisition by binning the pixels on the CCD sensor into 123 channels to reduce the measurement noise and amount of data to be stored and processed. The reflectance spectra were normalised with using a Spectralon™ reference panel (Labsphere, Inc., North Sutton, NH, USA). The panel was attached on the Sensei system so that the outermost 10 pixels of the line camera measured its reflectance during every spectrum collection operation.

The integration of laser scanning data and spectral data was done by direct georeferencing based on the post-processed data of the inertial navigation system. The post-processing was done through Kalman filtering using Waypoint Inertial Explorer software and GPS base station data from Geotrim’s VRS network. Exact information about the accuracy of the data is not available as no control points were measured, but based on the error metrics (e.g., standard deviation and separation between forward and reverse solutions) it is assumed to be better than 10 cm. Additional information about the system and data processing can be found in Jaakkola *et al.* [1].

### 2.2. Test Area, Measurements, and Data Sets

**Figure 3.**
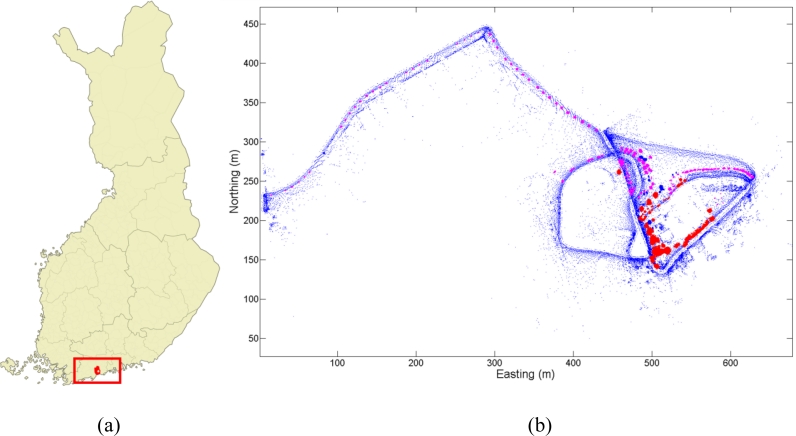
The Vanttila test area location is shown on a map of Finland (**a**) and an overview is presented of the test area (**b**). The overview image is drawn with the measured point cloud. The red and magenta objects are the determined trees used in classifications. Magenta objects have been included both in tree species classification and in coniferous-deciduous tree separation while red objects are only used in coniferous-deciduous tree separation. Blue areas represent the rest of the data (the map of Finland was retrieved from Wikipedia, created by user Care).

The test area used in the present study is located in southern Finland in the city of Espoo (60.209°N, 24.658°E); it consists of an experimental garden and the side of the street leading to it. The garden has over 200 tree and shrub specimens representing over 20 different species. The specimens represent a wide spectrum of species commonly used in Finnish parks and gardens. The studied specimens are mainly planted with small distances to each other and the study area was clear of understory. The test area overlay is shown in Figure 3.

The data were collected at the beginning of September in 2010. The date of the data collection was in the late summer in Finland and the leaves of trees were still green. The time of the data collection was after 9 o’clock in the morning. This meant that the sun’s zenith angle was close to 71° from nadir. This wide zenith angle meant that the shading effects were emphasized in the hyperspectral data making the classification more difficult. Peltoniemi *et al.* [52] and Suomalainen *et al.* [53] have studied the effects of directional light scattering in different ground types and in low vegetation. Their results showed that the difference between the minimum and the maximum reflectance measured from the same target can be over hundred percents depending on the viewing geometry. Therefore, it is likely that the measured hyperspectral data would have variations of at least of similar order in them.

The data were measured over a time span of 7 min and it consisted of five million laser points and 10,000 line spectra. The data were collected by driving along the street and the paths around and within the experimental garden.

## 3. Data Processing Steps, Classification Features, and Data Classification Procedures

Data processing steps, including specimen selection and determination, and the fusion of laser point cloud and hyperspectral data, are given in Section 3.1. All classification features and their extraction from specimen-wise fused data are presented in Section 3.2. Classification procedure used in specimen classification is described in Section 3.3.

### 3.1. Data Fusion Steps

Data processing and classification were done using the MATLAB 7.11 software (Mathworks, Natick, MA, USA). Individual trees and shrubs were determined manually from the laser point cloud. The determination was carried out in two steps: first, the tree specimen was outlined roughly from the bird-eye view. Second, the outlined tree specimen was determined more accurately repeating the outlining from different viewpoints in three dimensions. After all tree specimens were determined from the point cloud data they were fused with the hyperspectral line image data.

**Figure 4.**
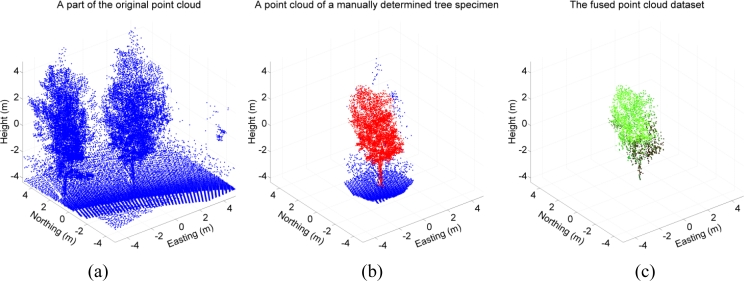
Data fusion process. **(a)** A part of the laser point cloud presenting a single tree specimen (*Sorbus hybrida*) set in the origin. **(b)** The same tree specimen after manual determination. The blue points represent the situation after 2D determination and the red points represent the outcome after an accurate 3D determination. These points are used in derivation of the height statistics of the tree specimen that were used in LiDAR-derived feature classification (Section 4.1). **(c)** A fused point cloud. Overlap between each determined laser point and hyperspectral pixels has been tested and all overlapping laser points have been given an individual colour spectrum. The average of all mapped spectra were used in hyperspectral classification (Section 4.2).

The data fusion process was as follow: The IMU locations were first interpolated by using the time stamps of the laser points. Then, the point cloud data were transformed into the Sensei inertial frame coordinates. After the transformation, possible overlap between the spectrometer line images and the laser points was tested first in the Sensei’s driving direction (horizontal). Next, the overlap test was repeated in the vertical direction for the pixels of each spectrometer line found within a laser spot. The colour values of every pixel located in a single laser spot were normalized against the reference spectrum measured from the Spectralon™ reference plate. The colour values of hyperspectral pixels were averaged if more than one pixel was found within a single laser point. An example of a manual tree specimen determination is shown in Figure 4.

The processed data were saved into a new data structure which contained the original laser point cloud and the hyperspectral data mapped on it. Spectral information could not be mapped on all laser points because the field-of-vision of the spectrometer did not extend the tree tops in the near range. The limited field-of-view of the line spectrometer did not have a significant effect on the data analysis as it could be detected only in the largest trees close to road in the data set. Also, spectral data was averaged over each tree specimen further diminishing the effect. Two datasets were formed from the fused data: one for coniferous and deciduous tree separation, comprising of 168 tree specimens, and the other for individual tree species classification comprising of 133 tree specimen representing 10 tree species. The dataset sizes differed from each other as the specimen numbers of some tree species was below five, which was set as the threshold value. Another reason for the dataset discrepancy was that in some cases two or more coniferous or deciduous tree specimens were growing in the immediate proximity of each other, thus preventing accurate determination of the species. The examined tree species and their numbers in the dataset are listed in Table 1. It should be noted that the number of tree specimens of different species is unequal, which is likely to have an effect during the classification procedure.

### 3.2. Feature Extraction for Classifications

Certain Light Detection and Ranging LiDAR-derived features and hyperspectral features were extracted from the fused tree datasets for classification. In all 34 LiDAR-derived features and 123 hyperspectral features were calculated for each tree specimen point cloud.

The classification feature extraction process followed the one presented in Puttonen *et al.* [54]. The LiDAR-derived features, were calculated from the height distributions of the laser-scanned point clouds of each determined tree specimen. The point cloud height distributions were used in feature extraction as the viewing geometry of the measurement was horizontal. Thus, an accurate vertical profile of each tree specimen was collected as there was little or no occlusion in the determined data. Table 2 presents all of the LiDAR-derived features used in the classification. The first twenty LiDAR-derived features described the proportions of laser hits found within the normalized height fractions in a tree specimen. Skewness and kurtosis were the third and the fourth standardized moments of the laser point height distribution. Maximum tree height was defined as the entire length of a tree specimen. The mean height of a single tree specimen was calculated from all laser hits coming from that tree specimen. The height coefficient of variation was the standard deviation of a single-tree-laser-point-cloud height that was divided by its mean height. The use of LiDAR-derived features describing the ratios of point cloud height distribution over a selected normalized height threshold (PR) and the height quantiles (hq) have been inspired by the area-based study of Naesset and Gobakken [55], who used canopy height distributions obtained from small-footprint airborne laser scanner data to estimate forest growth in both young and mature boreal forests. They found out that canopy height parameters that were obtained from lower, intermediate and the top parts of trees were the best estimators. Furthermore, the first return pulse information was better suited for forest growth estimation than the last pulse information in their study.

The hyperspectral classification features were formed by averaging the intensities of all of the measured fused points of an individual tree specimen point cloud. Each tree specimen point cloud was described using the total of 123 spectral values after averaging. The intensity value averaging reduced the total amount of the data significantly. Also, the large single point reflectance variations still present in the data after intensity normalization were further attenuated in conjunction with intensity averaging. However, intensity averaging over a whole tree specimen meant that the information about the directional lighting effects and the spectral differences in different parts of the tree specimen were lost. This did most likely have a negative effect on the overall classification accuracies.

### 3.3. Classification Procedure

The extracted features were first used for more general classification between deciduous and coniferous trees and then separately for individual tree species classification. The classifications were performed using the LibSVM software package developed by Chang and Lin [56]. LibSVM is a Support Vector Machine (SVM) classifier [57]. It uses an improved Sequential Minimal Optimization algorithm in SVM training [58]. SVMs have been successfully applied in several studies for conducting various remote sensing classifications [30,59,60].

The LibSVM classification was done following the guideline given in Chang and Lin [56]. First, the values of the features chosen for classification were scaled between −1 and 1. Scaling is necessary to avoid possible numerical problems. Another reason for the scaling is to set the different classification features on an equal level in regards to one with another. A Radial Basis Function (RBF) was used as the kernel. The optimization of the two kernel parameters was carried out by cross-validating the data several times while changing the kernel parameter values by several orders of magnitude during the process.

All classifications and SVM kernel parameter optimizations were done applying the leave-one-out cross-validation (LOOCV) setup. Each tree specimen was classified using the rest of the specimens as a training set for a classifier. Individual specimen results were then collected together to provide the overall result. The LOOCV setup was applied despite it being computationally intensive as the total number of classified trees was small (see Table 1).

The classification feature testing procedure has been presented in Puttonen *et al.* [54], where three tree species were classified in laboratory conditions. Both the separation of coniferous and deciduous species and the classifications of individual tree species were carried out several times while systematically testing the classification features and their combinations. All LiDAR-derived and hyperspectral features were used one at time in the first classifications. All possible feature pairs, consisting of the LiDAR-derived and the hyperspectral features, were also tested in the same manner.

Finally, a selected set of both LiDAR-derived and hyperspectral feature pairs were combined together to make new feature quadruples for a new classification round. The feature quadruples were selected by choosing the best-performing 10% from both LiDAR-derived and hyperspectral feature pairs. This selection was carried out to reduce the total amount of possible feature combinations.

### 3.4. Validation of the Selected Classifier and the Feature Selection

The validity of the classifier and feature selection was tested in three ways. First, the classification was repeated for the best single, paired, and combined classification features and their sets with a linear discriminant classifier. The linear discriminant classifier was chosen as a reference to justify the use of a computationally more complex SVM classifier. The second method of testing feature selection was performed by conducting the classification using all of the LiDAR-derived features and hyperspectral features at the same time in the classification. This was done to see if there was any clear improvement in the overall classification accuracy. If the classification results, with all possible features available in the study, did not yield the best outcome with a wide margin compared to smaller feature subsets, then their use as a whole feature set was not recommended. The reason for not to use a large feature set with a size close to or larger than the specimen number was that the obtained result became susceptible to overfitting. In an overfitting situation, the classifier classifies given training data with high accuracy, and this includes fluctuations and noise. Thus, its predictive power for the whole dataset is weak [61]. Furthermore, the classification efficiency is improved when as minimal number of classification parameters as possible are used to obtain an accurate classification result. The classification efficiency improves as less data and processing time are needed.

And third, we tested the validation of the classification feature selection by using a forward selection method where the classification was performed in iterative steps. The forward selection approach was chosen as the third validation method as it has been suggested in literature as a good starting point when launching a new classification project [62]. First, the best-tested LiDAR-derived or hyperspectral feature was selected. This was followed by repeating the classification with all of the other shape and hyperspectral features paired with the best one. The best feature pair was then selected and it was used with the rest of the features in the following iteration round. A total of four iterations with different LiDAR-derived feature and hyperspectral feature combinations were tested.

## 4. Classification Results

The classification results are presented in five parts: (i) The overall results of both single and paired LiDAR-derived features; (ii) The overall results of both single and paired hyperspectral features; (iii) The comparison of the results obtained from the LiDAR-derived and the hyperspectral features; (iv) The detailed classification results of combined feature quadruples consisting of two hyperspectral features at different wavelengths and of two LiDAR-derived features; (v) Different classification feature selection methods are compared in order to validate the presented results.

### 4.1. Classification Results with LiDAR-Derived Features

The classification was performed with 34 LiDAR-derived features and with all possible (561) LiDAR-derived feature pairs formed out of them. The best classification result for coniferous and deciduous tree separation with a single LiDAR-derived feature was 84.5% and it was obtained with a LiDAR-derived feature describing the relative number of points over the midpoint of the normalized tree height (PR, h_N_ > 0.5 in Table 2). The best paired classification result was 90.5% and it was obtained with LiDAR-derived features that were the relative number of points over 10% of the normalized tree height (PR, h_N_ > 0.5) and the kurtosis of the point cloud height distribution. The classification result with all LiDAR-derived features was 89.9%.

It should be borne in mind that the measurements were conducted in an experimental garden where the specimen trees had not yet reached full maturity. This caused additional variance in the shape-based classification between deciduous and coniferous trees. Also, it should be emphasized here that the features, both LiDAR-derived and hyperspectral, specifically labeled in the text were not the only features giving the best classification results. There were several other possible features that achieved the same classification accuracy. This was especially the case with the paired features and with the feature quadruples.

The tree species were classified in a similar manner. The best classification results for single and paired LiDAR-derived features were 51.1% and 65.4%. The best found single feature described the relative number of points over 40% of the normalized tree height (PR, h_N_ > 0.5). The best feature pair consisted of the relative number of points below 33% of the normalized tree height (PR, h_N_ < 0.33) and of the 20% height quantile (hq20). The species-wise classification result with all LiDAR-derived features was 67.7%.

A fraction of the best-performing LiDAR-derived feature pairs were selected for the four-feature classification. The number of LiDAR-derived feature pairs selected for coniferous and deciduous tree separation was 73 and these represented 13.0% of all pairs. For tree species classification, the corresponding number was 62 pairs from a total of 561 (11.1%). The mean classification performance of the selected LiDAR-derived feature pairs and their standard deviations are shown in Tables 3 and 4.

The LiDAR-derived features separate coniferous trees from deciduous trees with a relatively high accuracy. The best single LiDAR-derived features were able to achieve this with an accuracy of over 80%. The best performing single LiDAR-derived features were the relative number of points over 10% of the normalized tree height (PR, h_N_ > 0.1), the relative number of points over 40% of the normalized tree height (PR, h_N_ > 0.4), and the relative number of points over the midpoint of the normalized tree height (PR, h_N_ > 0.5). The best-performing LiDAR-derived features in this case are all point ratios over a certain height threshold. Thus, the result implies that there was a systematic shape difference between coniferous and deciduous trees in the data. The result is logical as most of the coniferous species included in the data had a conical shape (especially young specimens), while the deciduous tree species had a more clear division between their canopies and trunks.

The LiDAR-derived feature pair results were more accurate than the results obtained with individual LiDAR-derived features. Especially, deciduous trees were separated on average with an accuracy of over 90% within the selected pair set (Table 3). The standard deviation was below 3%, which implied that the results were consistent. The result obtained using all of the LiDAR-derived features in the classification showed that no separation improvement was gained when its results were compared with the performance of the best single and paired LiDAR-derived features.

The LiDAR-derived tree species classification results showed a more obvious accuracy improvement between single and paired LiDAR-derived features when compared to the coniferous-deciduous separation results. This was to be expected as the number of different classes rose to ten from previous two. However, Table 4 shows a clear ambivalence in species classification results: Three of the tree species have been classified on average with an accuracy of over 80%, namely *Sorbus americana*, *Quercus robur*, and *Abies sibirica*. These were also the three most numerous species in the dataset. The classification accuracies for the other species ranged from 0% to 60.2% in the selected feature pair sets. The main constituents of the classification errors were misclassifications within the genus *Sorbus* (Table 1) and with *Quercus robur*. Other misclassifications occurred between the other deciduous species and between the two coniferous species. The misclassifications occurred systematically, which means that the classifier favoured the species providing more specimens.

### 4.2. Classification Results with Hyperspectral Features

The classification was performed with 123 different hyperspectral feature values and with all possible hyperspectral feature pairs (7,503) formed from them. The best classification result for coniferous and deciduous tree separation with a single hyperspectral feature was obtained with a wavelength channel centered at 988 nm and was 79.2%. The best paired classification result was 90.5%. The result was obtained with wavelength channels centered at 932 nm and at 994 nm, whereas the classification accuracy utilizing the full spectrum (all of the hyperspectral features) was 93.2%. All listed hyperspectral features with the best prediction power are located in the infra-red (IR) part of the spectrum. This is expected as the deciduous tree species in this study are brighter in general in the IR region than the coniferous species.

The best tree species classification results for single and paired hyperspectral features were 43.6% and 62.4%. The best single hyperspectral feature was the wavelength channel centered at 954 nm) and the best hyperspectral feature pair was consisted of wavelength channels centered at 489 nm and at 781 nm. The species-wise classification result with full spectrum was 66.9%. The best single hyperspectral feature was located again in the IR region. The best hyperspectral feature pair, however, was selected around 700 nm where vegetation spectrum is known to have significant brightening. Thus, this implies that for several different tree species the spectral information from the IR region alone is not enough to distinguish tree species from each other. As in Section 4.1, a fraction of the best performing hyperspectral-feature pairs were selected for the four-feature classification. The number of hyperspectral-feature pairs selected for coniferous and deciduous tree separation was 754, which represented 10.0% of all pairs. The corresponding numbers were 786 pairs of a total of 7,503 (10.5%) for the tree-species classification. The mean classification performance of the selected hyperspectral feature pairs and their standard deviations are shown in the Tables 3 and 4. The coniferous and deciduous tree separation succeeded with classification accuracy similar to that of LiDAR-derived shape-feature-based case. Deciduous tree separation succeeded better than in the shape-based case, but the coniferous tree separation result was several percentage points lower than in the shape-based separation. The total coniferous and deciduous tree separation result was on par with the shape-based case. The standard deviation within the selected hyperspectral feature set was low, below 3% in the overall classification result. The use of all hyperspectral features yielded a better overall separation result when compared to the average results of the hyperspectral feature pairs. This means that the redundancy between spectral channels was relatively small.

### 4.3. Comparison between the LiDAR-Derived and the Hyperspectral Classification Results

The results of the classification using hyperspectral and LiDAR-derived features differed in a couple of ways: First of all, only one species, *Quercus robur*, was classified with an average accuracy of over 80% when using the hyperspectral features. Another issue is that the species classification performance differed from the shape-based classification. For example, *Sorbus intermedia* was fully confused with the other *Sorbus* species in the shape-based classification was subsequently classified correctly in almost half of the cases. On the other hand, several species received significantly more incorrect results when compared to the shape-based classification: The classification accuracy of *Sorbus americana* dropped almost 50% points due to it being confused with the other *Sorbus* species. Also *Ulmus glabra*, *Picea pungens*, and *Abies sibirica* were misclassified more often than other species in shape-based classification. The average overall classification performance of the selected hyperspectral feature pairs was close to 5% points lower than in the shape-based classification. Additionally, the full spectrum classification gave better results in species classification than did the selected hyperspectral feature pairs. The performance difference between the average of the selected hyperspectral feature pairs and the full spectrum was over 10% points, which would appear to imply that using more than two hyperspectral features in tree species classification would be better for this dataset. However, when coupled with LiDAR-derived shape features, even two separate hyperspectral features could yield significant improvements in the overall species classification. Also, in reality, where training the classifier is based on a practical number of reference trees, the practical results using several features more will not yield significantly higher accuracies.

### 4.4. Classification Results Based on both the LiDAR-Derived and the Hyperspectral Features

The fused data classification was carried out by forming feature quadruples from the best-performing feature pairs selected in Sections 4.1 and 4.2. Each feature quadruple consisted of one shape-feature pair and one hyperspectral-feature pair. The total number of combined feature quadruples was 55,042 unique sets for the coniferous and deciduous tree separation, and 48,732 unique feature quadruples for the tree species classification. The total number of selected feature quadruples was limited to constrain the processing time within practical limits.

The best overall coniferous and deciduous tree separation result was 95.8%. The average separation accuracy of feature quadruples was 90.9% (Table 3). The averaged accuracy was a few percentage points higher than the one obtained using only the LiDAR-derived shape or hyperspectral feature pairs, but no significant separation advantage was gained. The overall separation accuracy improved mainly as conifers were detected more effectively than in the earlier pair-wise separations. The standard deviation of the overall result was under 3% and this implies that the feature selection within this group should have yielded close to 90% separation accuracy in most cases.

The best tree species classification resulted in 83.5% accuracy, while the average classification result for all 48,732 tested feature quadruples was 72.2% with a standard deviation of 9.0% (Table 4). The average result shows that combining well-performing feature pairs into new quadruples results in a further improvement in overall tree-species classification accuracy.

Three species, *Sorbus aria*, *Ulmus glabra*, and *Picea pungens* were still confused with other species, which resulted in their species-wise accuracy falling below 40%. On the other hand, the classification accuracy of *Sorbus hybrida* improved significantly, up to 77.7% when compared to pair-wise classification accuracy (LiDAR 22.6%, hyperspectral 33.6%). The improvement in the overall classification accuracy was also significant when compared to the pair-wise results; this supports the use of combined feature sets in species-wise classification.

Table 5 contains the error matrix of the best species-wise result obtained with a feature quadruple consisting of the LiDAR-derived features that were the 90% height quantile (hq90) and the mean height of a single tree specimen, and the hyperspectral features that were channels centered at 428 nm and at 982 nm. The table shows that the species represented by the most specimens were classified correctly or close to correctly. There was clear confusion between the species of the genus *Sorbus*. On the other hand, only a few *Sorbus* specimens were misclassified as other tree species. All *Quercus robur* and *Salix fragilis* specimens were classified correctly. Deciduous *Sorbus aria* and coniferous *Picea pungens* were classified with the lowest accuracies. They were also the species represented by the smallest number of specimens. *Sorbus aria* was confused in three cases with the *Sorbus aucuparia*, which is a member of the same genus. *Picea pungens* was misclassified as being several different species. Table 4 shows that the classification of *Picea pungens* was based almost completely on the LiDAR-derived shape information. This could mean that no reliable spectral data were collected on them. Possible reason for non-reliable spectra for these trees could have been poor local lighting conditions caused by shading and the sun being located close to the horizon at the time. Another possible reason is that the reference plate has been illuminated at the time while the tree specimen has been in a shade at the time. This would have resulted in very dark normalized spectra.

### 4.5. Classification Method Comparisons

The classification efficiency between LibSVM and a linear discriminant analyser (LDA) was tested to justify the use of the more complex and computationally more intensive SVM. The testing was performed using the best feature pairs and quadruples in comparison. The comparison results are shown in Table 6. Overall, the results showed a clear difference between the classification accuracies in favour of the LibSVM. The LibSVM outperformed the LDA by over 5% points in all of the paired test cases. Moreover, the classification performance of the LDA is more sensitive to the number of classes determined and the number of classification features than the LibSVM. However, the best feature quadruples resulted in LDA results show that for coniferous and deciduous tree separation almost equal classification accuracy (94.1%) could be achieved when compared with the best SMV result. Also, while there was still a clear difference, the best tree species classification result with the LDA (79.7%) was relatively close to the best obtained SVM classification result. Thus, the best results imply that the feature selection works for both types of classifiers.

**Figure 5.**
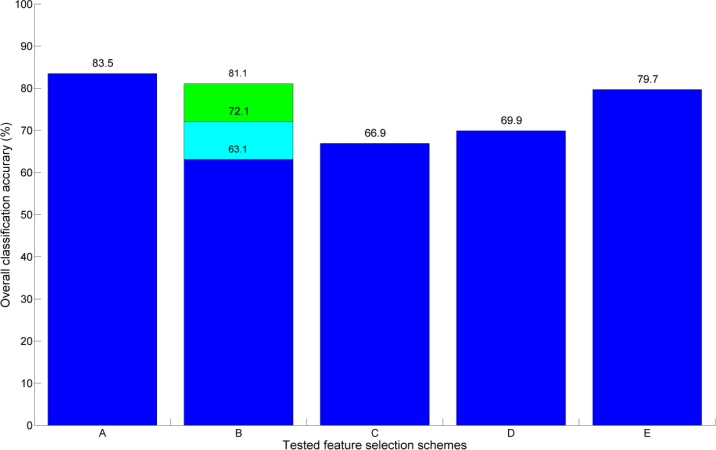
Classification accuracy comparison between different four-feature classification parameter sets. Bars **A** and **B** were obtained from the feature quadruples formed in Section 4.3. Bar **A** shows the best classification result of the feature quadruples while bar **B** represents their average classification result and its standard deviation (see Table 4). Bar **C** is the overall classification result obtained with four forward-selected hyperspectral features. Bar **D** is the overall classification result of four forward-selected shape features. Bar **E** is the best overall classification result obtained with two forward-selected LiDAR-derived shape features and two hyperspectral features.

Another classification efficiency test was also performed to test the effect of different feature selection methods on overall tree-species classification accuracy. In the test, three different types of forward-selected four-feature comparison sets were formed: One set with four LiDAR-derived shape features, one with four hyperspectral features, and a feature set with two shape features and two hyperspectral features giving the best overall classification result. The forward selection test was chosen, because its implementation is straightforward and it has a low computational complexity. The comparison results are shown in Figure 5. The forward-selection test was performed only for the species classification.

The results showed that the best feature quadruple (case (**A)** in Figure 5) of the tested 48,732 feature quadruples gave the highest overall classification accuracy. Moreover, the average classification result of all of the tested feature quadruples (**B**) exhibited higher classification performance than the four-feature sets based only on LiDAR-derived shape (**C**) or hyperspectral features (**D**). This result emphasizes the efficiency of feature quadruples combined from different types of data sources over the feature sets that had been derived using single sensor data.

However, the best forward-selected four-feature sets, both the LiDAR-derived (**C**) and the hyperspectral one (**D**), yielded significantly better overall classification results than their paired counterparts (see Table 4). The margin between the classification accuracies was several percentage points. This was to be expected as the number of classification features doubled from what it was with the paired cases. Moreover, these two results also show that the use of more than four features of the same data type does not improve the classification accuracy for these data. This is seen when comparing them against the classification result obtained with all of the LiDAR-derived shape features (67.7%) and against the result obtained with all of the hyperspectral features (66.9%). The reasons for not gaining further improvement in classification accuracy were most likely in the relatively high variation within the data as well as the redundancy within the classification features themselves.

The best result was obtained with the mixed forward-selected four-feature set (**E**) that classified tree species with an overall accuracy of 79.7%. The average classification accuracy of the tested six forward-selected four-feature sets was 77.3% and their standard deviation was 1.5%. This implies that the selection order of the forward-selected four-feature set does not have a significant role in the outcome of the classification. However, the best results were obtained when at least one LiDAR-derived shape feature was selected in the first iteration. The average overall classification score within all of the tested forward-selected four-feature sets and its low variation suggested that the forward-selected feature sets provided a straightforward way to achieve relatively high classification accuracy. However, the best overall classification accuracy obtained with the feature set (**A**) was over five percentage points higher than the average of the forward-selected four-feature sets. This implies that forward selection of the features limits the chances of finding the best possible classification feature combination as it locks the previous features during iteration.

Heinzel *et al.* [32] have reported similar results with forward selection when they classified tree species using features derived from ALS waveform data. There were clear classification accuracy improvements in their study during the first forward selected iterations. However, the classification accuracy did not significantly change when more features were included in classification.

## 5. Conclusions

The study presents the first results of using mobile laser scanning and hyperspectral tree data in tree species classification. Tree species classification and the separation of coniferous and deciduous trees were performed in a city experimental garden in the City of Espoo, Finland. The results showed that a fused data set consisting of LiDAR-derived and hyperspectral features outperformed single-source data sets by a significant margin. The best overall coniferous and deciduous tree separation result was 95.8% when two LiDAR-derived shape and two hyperspectral features were applied using a Support Vector Machine (SVM) as a classifier. The corresponding best tree species classification result including 10 species in the analysis was 83.5%. The results were obtained using a low number of predictors to give a more realistic and practical view of the potential of the data.

The SVM is a powerful classification tool. Thus, it is difficult to have an idea of the complexity of the classification task when it is used alone. Therefore, we used a Linear Discriminant Analyser (LDA) as a reference. The results of the LDA indicated how well the different classes were separated in the feature space and thus what was the minimum level of separation to be expected when other classifiers were used. The results obtained with the LDA and the SVM showed that the overall classification results could be improved with a more sophisticated classifier when the number of classification features was limited to a few and several classes were classified.

The best obtained classification accuracy for these data is close to a level where its use could be considered for larger scale studies. Before this however, there is a need for more comprehensive result validation with a more even tree specimen variety. Also, more studies that cover longer time spans are needed to detect phenology-related changes in trees. Furthermore, the workflow optimization and the automatization level of the feature extraction both need improvement before data processing on an operational level will be feasible. Overall, it should be kept in mind that the suitability of this type of data and the possibly obtainable classification accuracy are always application-specific and that their range needs to be considered separately for each individual study case.The lighting conditions were observed to play an important role in the hyperspectral response. Thus, it was found to be necessary to take directional lighting factors better into account in order to further improve the classification accuracy in future analyses. A possible solution for better hyperspectral detection might be in active multi-wavelength laser systems that are capable of simultaneous range and intensity detection [63]. They would significantly simplify the detection geometry and negate most of the issues related to diffuse lighting and shading effects. Overall, mobile mapping is seen to be a feasible application of technology utilizing directional lighting effects.

The results presented here are also among the first of their kind. Therefore, direct comparison with other similar studies is not possible. However, corresponding studies have been done with fused data where ALS data and hyperspectral data have been combined together. Dalponte *et al.* [30] reported on classification results for 23 land-cover classes. The first feature set consisting of 40 hyperspectral features gave equal explanatory power compared to the use of a single feature from the ALS, *i.e.*, the elevation of the first return. They also observed that for some dominant classes accuracy was of the order of 85%–90%, whereas, for the minority classes, a dramatic decrease in accuracy was observed when the data were analyzed with SVM class-by-class accuracy.

In another study, Asner *et al.* [28] reported of the detection of three invasive tree species in Hawaiian rainforests. They used LiDAR data in shadow removal and tree-crown quantification. Then, they proceeded to collect the full-range hyperspectra of over 200 spectral channels of the tree crowns and analysed the data applying a spectral mixture in several stages. The results showed that the invasive species could be detected within ∼2 m^2^ and ∼7 m^2^ minimum canopy thresholds with error rates of less than 18.6% and 6.8%.

The classification accuracies of the results presented in this study are comparable. However, several differences do apply to the data and their collection. The main difference between this study and the others is in the different measurement geometry, which results in significantly varying directional lighting conditions. The lighting conditions in mobile terrestrial survey are challenging as the measurements are sometimes done towards and away from the sun. Directional lighting effects, such as bidirectional reflectance [64], and their calibration have to be considered in future, as these effects are more severe in mobile than in airborne use. In addition to the directional lighting effects, the viewing geometry is sensitive to the lateral occlusion. Therefore, the viewing depth in horizontal direction is limited in the case of densely packed vegetation. This problem can be diminished if waveform data is available.

However, the point density of the study was higher than in the previous examples, a low-cost laser scanner and spectrometer were applied, and only a small number of classification predictors were used. The presented system and classification method can be applied in several ways in the future. For example, city authorities need information on park and road-side trees for planning and management purposes. Mobile mapping methods could position trees automatically while collecting other important main stand attributes, such as stem diameters and volumes, and tree height and tree species. Tree health monitoring could also be a possible new application area. Also, the classification results presented here were accurate enough to imply that the presented system should be also capable of collecting data for more general object recognition.

In near future, we anticipate that data collected with MLS systems will find increasing use in urban planning. Another application of mobile mapping systems could involve the creation of virtual environments when integrated with ALS and aerial images. Recently, the authorities of the City of Helsinki in Finland have shown interest in presenting virtual models of eastern Helsinki in computer games to enable the public to take part in the planning of new building areas. This would allow more citizens to participate in the planning and voting for different solutions, and this would give the public more powers of influence in urban decision-making. Consequently, the work needed in urban planning would become easier.

## Figures and Tables

**Table 1.** Classified tree species and their specimen numbers. All listed tree specimen were used in coniferous-deciduous tree separation. The tree species with the specimen number in bold were also used in individual tree species classification. Five of the specimens labelled as unidentified were deciduous and two specimens were coniferous.

**Index**	**Common name**	**Latin name**	**Number of specimens**
**1**	Finnish Whitebeam	*Sorbus hybrida*	**6**
**2**	Swedish Whitebeam	*Sorbus intermedia*	**8**
**3**	European Rowan	*Sorbus aucuparia*	**21**
**4**	Common Whitebeam	*Sorbus aria*	**9**
**5**	American Mountain-ash	*Sorbus americana*	**23**
**6**	Pedunculate Oak	*Quercus robur*	**18**
**7**	Norway Maple	*Acer platanoides*	4
**8**	Apple	*Malus domestica*	3
**9**	Hungarian Lilac	*Syringa josikea*	1
**10**	Common Alder	*Alnus glutinosa*	4
**11**	Camperdown Elm	*Ulmus glabra camperdownii*	**5**
**12**	Crack Willow	*Salix fragilis, ‘Bullata’*	**8**
**13**	Colorado Blue Spruce	*Picea pungens, ‘Iseli Fastigiate’*	**5**
**14**	Black Spruce	*Picea mariana*	4
**15**	White Fir	*Abies concolor*	2
**16**	Siberian Fir	*Abies sibirica*	**30**
**17**	Balsam Fir	*Abies balsamea*	2
**18**	Common Juniper	*Juniperus communis*	2
**19**	European Yew	*Taxus baccata*	2
**20**	Northern Whitecedar	*Thuja occidentalis, ‘Danica’*	1
**21**	Common Douglas-fir	*Pseudotsuga menziesii*	1
**22**	Silver Birch	*Betula pendula*	1
**23**	Scots Pine	*Pinus sylvestris*	1
*	Unidentified tree species	*--*	7
	Total number of trees		168 (**133**)

**Table 2.** LiDAR-derived tree point cloud height distribution features and their descriptions.

**LiDAR-derived feature index**		

PR, h_N_ < 0.33	PR, h_N_ > 0.2	hq 30
PR, 0.33 < h_N_ < 0.67	PR, h_N_ > 0.3	hq 40
PR, h_N_ > 0.67	PR, h_N_ > 0.4	hq 50
PR, 0.1 < h_N_ < 0.2	PR, h_N_ > 0.5	hq 60
PR, 0.2 < h_N_ < 0.3	PR, h_N_ > 0.6	hq 70
PR, 0.3 < h_N_ < 0.4	PR, h_N_ > 0.7	hq 80
PR, 0.4 < h_N_ < 0.5	PR, h_N_ > 0.8	hq 90
PR, 0.5 < h_N_ < 0.6	PR, h_N_ > 0.9	Max
PR, 0.6 < h_N_ < 0.7	Skewness	Mean
PR, 0.7 < h_N_ < 0.8	Kurtosis	CV
PR, 0.8 < h_N_ < 0.9	hq 10	
PR, h_N_ > 0.1	hq 20	

PR(h_N_) = Proportion of laser hits within a shown normalized height interval in a tree specimen; Skewness = Skewness of the height distribution of a tree specimen point cloud; Kurtosis = Kurtosis of the height distribution of a tree specimen point cloud; n:th hq = n:th height quantile in percents, from the base of the tree; Max = Maximum height of the laser hits in a tree specimen; Mean = Mean height of the laser hits in a tree specimen; CV = Coefficient of variation

**Table 3.** The mean deciduous and coniferous tree separation results of the selected LiDAR-derived and hyperspectral feature pairs, and all of the feature quadruples thus formed. The number of the LiDAR-derived feature pairs was 73, the number of hyperspectral feature pairs was 754, and the number of selected feature quadruples was 55,042.

	
	**LiDAR-derived feature pairs**		**Hyperspectral feature pairs**		**Feature quadruples (2 LiDAR and 2 hyperspectral features)**	

	**Mean %**	**Std %**	**Mean %**	**Std %**	**Mean %**	**Std %**

**Deciduous**	92.3	2.7	93.9	2.0	94.1	1.8
**Coniferous**	75.0	5.8	68.9	4.8	83.6	3.6
---	---	---	---	---	---	---
**Total**	86.9	3.6	86.2	2.9	90.9	2.4

**Table 4.** The average species classification results of the selected LiDAR-derived and hyperspectral feature pairs, and all of the feature quadruples thus formed. The species indexing is given in Table 1. The number of the LiDAR-derived feature pairs was 73, the number of hyperspectral feature pairs was 786, and the number of selected feature quadruples was 48,732.

	
	**LiDAR-derived feature pairs**		**Hyperspectral feature pairs**		**Feature quadruples (2 LiDAR and 2 hyperspectral features)**	

**Species index**	**Mean %**	**Std %**	**Mean %**	**Std %**	**Mean %**	**Std %**

*Sorbus hybrida*	22.6	20.7	33.6	22.8	77.7	12.6
*Sorbus intermedia*	0.0	0.0	48.2	6.8	53.0	11.0
*Sorbus aucuparia*	39.5	11.3	60.4	12.1	57.2	9.5
*Sorbus aria*	12.9	13.7	25.6	18.9	14.8	17.5
*Sorbus americana*	83.9	6.1	37.0	14.5	84.3	7.0
*Quercus robur*	83.6	4.3	93.3	6.7	98.3	3.4
*Ulmus glabra camperdownii*	45.2	33.4	28.8	19.3	34.1	29.0
*Salix fragilis*	61.3	13.5	77.2	15.7	70.2	16.5
*Picea pungens*	16.1	16.5	0.0	0.7	17.3	15.6
*Abies sibirica*	93.2	4.9	72.0	6.4	95.1	3.3
---	---	---	---	---	---	---
**Total**	61.0	9.1	56.7	11.2	72.2	9.0

**Table 5.** The error matrix of the species-wise classification result of the best selected feature quadruple (LiDAR-derived features were the 90% height quantile (hq90)andthe mean height of a single tree specimen, and hyperspectral features that were the channels centered at 428 nm and at 982 nm). Bolded numbers in the diagonal are the numbers of correctly classified tree specimens. All classification accuracies are given in percents.

**Species Index**	***Sorbus hybrida***	***Sorbus intermedia***	***Sorbus aucuparia***	***Sorbus aria***	***Sorbus americana***	***Quercus robur***	***Ulmus glabra camperdownii***	***Salix fragilis***	***Picea pungens***	***Abies sibirica***	**User accuracy**
*Sorbus hybrida*	**5**	1	0	0	0	0	0	0	0	1	71.4
*Sorbus intermedia*	0	**5**	2	0	0	0	0	0	0	0	71.4
*Sorbus aucuparia*	0	1	**15**	3	1	0	0	0	0	0	75.0
*Sorbus aria*	0	0	3	**5**	0	0	0	0	0	0	62.5
*Sorbus americana*	1	0	0	0	**22**	0	0	0	0	0	95.7
*Quercus robur*	0	0	0	0	0	**18**	0	0	0	0	100.0
*Ulmus glabra camperdownii*	0	0	0	0	0	0	**3**	0	1	0	75.0
*Salix fragilis*	0	0	0	0	0	0	2	**8**	1	0	72.7
*Picea pungens*	0	0	0	1	0	0	0	0	**1**	0	50.0
*Abies sibirica*	0	1	1	0	0	0	0	0	2	**29**	87.9
											**Total accuracy**
**Producer accuracy**	83.3	62.5	71.4	55.6	95.7	100.0	60.0	100.0	20.0	96.7	**83.5**

**Table 6.** Classification accuracy comparison between the results of a Linear Discriminant Analyser (LDA) and the LibSVM. The best result of each case is reported.

	
	**LDA**	**LibSVM**

	**%**	**%**

**Coniferous-deciduous separation**	---	---
LiDAR-derived feature pair	86.3	90.5
Hyperspectral feature pair	81.0	90.5
Fused feature quadruple	94.1	95.8
**Tree species classification**	---	---
LiDAR-derived feature pair	54.9	65.4
Hyperspectral feature pair	54.1	62.4
Fused feature quadruple	79.7	83.5
